# Advantages of pooling of human bone marrow-derived mesenchymal stromal cells from different donors versus single-donor MSCs

**DOI:** 10.1038/s41598-024-62544-8

**Published:** 2024-06-02

**Authors:** Suresh Kannan, S. Gokul Krishna, Pawan Kumar Gupta, Uday Kumar Kolkundkar

**Affiliations:** Stempeutics Research Pvt Ltd, 3rd Floor, Manipal Hospitals Whitefield Pvt. Ltd., #143, EPIP Industrial Area, ITPL Main Road, Bangalore, Karnataka 560 048 India

**Keywords:** Mesenchymal stromal cells, Pooling, Heterogeneity, Secretome, Immunosuppression, Cell biology, Stem cells

## Abstract

Mesenchymal stromal cells (MSC) from adult bone marrow are the most commonly used cells in clinical trials. MSCs from single donors are the preferred starting material but suffer from a major setback of being heterogeneous that results in unpredictable and inconsistent clinical outcomes. To overcome this, we developed a method of pooling MSCs from different donors and created cell banks to cater clinical needs. Initially, the master cell banks (MCBs) were created at passage 1 (P1) from the bone marrow MSCs isolated from of nine different donors. At this stage, MCBs from three different donors were mixed in equal proportion and expanded till P3 to create working cell banks. Further, the pooled cells and individual donor MSCs were expanded till P5 and cryopreserved and extensively characterised. There was a large heterogeneity among the individual donor MSCs in terms of growth kinetics (90% Coefficient of variation (CV) for cell yield and 44% CV for population doubling time at P5), immunosuppressive ability (30% CV at 1:1 and 300% CV at 1:10 ratio), and the angiogenic factor secretion potential (20% CV for VEGF and71% CV for SDF-1). Comparatively, the pooled cells have more stable profiles (60% CV for cell yield and 7% CV for population doubling time at P5) and exhibit better immunosuppressive ability (15% CV at 1:1 and 32% CV at 1:10 ratio ) and consistent secretion of angiogenic factors (16% CV for VEGF and 51% CV for SDF-1). Further pooling does not compromise the trilineage differentiation capacity or phenotypic marker expression of the MSCs. The senescence and in vitro tumourigenicity characteristics of the pooled cells are also similar to those of individual donor MSCs. We conclude that pooling of MSCs from three different donors reduces heterogeneity among individual donors and produces MSCs with a consistent secretion and higher immunosuppressive profile.

## Introduction

Mesenchymal stromal cells (MSCs) are the most commonly used cells in the fields of cell therapy and regenerative medicine. MSCs are found in almost all the organs of the human body including bone marrow, adipose tissue, dental tissues, muscle, peripheral blood, etc.^1^ and play a major role in tissue homeostasis. According to the International Society for Cellular Therapy (ISCT) guidelines, MSCs are plastic adherent, spindle-shaped cells that express CD73, CD90, and CD105 while lacking expression of CD45, CD34, CD14 or CD11b, CD79α or CD19, and HLA-DR surface antigens^2^. They can differentiate into the cells of adipo, osteo, and chondro lineages. However, under proper conditions, MSCs also have the ability to differentiate into non-mesodermal lineages like cardiomyocytes, neuronal cells, epithelial cells, etc. Apart from these basic qualities, MSCs secrete a myriad of growth factors, cytokines, and other signaling molecules. In the clinical setting, MSCs exert their action either by differentiating into the cells of interest or by modifying the microenvironment by paracrine secretions. Now, it is widely accepted that the latter mechanism is the potential way by which MSCs act^3^.

There are different clinical trials that prove the safety and effectiveness of using MSCs in various acute and chronic indications such as critical limb ischemia, osteoarthritis, sepsis, myocardial infarction, and autoimmune disorders, among others^4–7^. The most commonly used MSCs are from bone marrow (BM), adipose tissue (AT), and perinatal tissues including the umbilical cord. Though the basic characteristics of the MSCs remain the same among the different sources, there exists a variation in terms of their ability to secrete various growth factors and immunomodulatory characteristics that decides the clinical outcome^8^.

Bone marrow-derived MSCs (BMMSCs) have the advantage and are the preferred choice of MSCs due to the ease of extraction and culturing, accessibility, and ability for both autologous and allogenic treatment. Conventionally BMMSCs were isolated from individual donors, with the culture expanded in either serum-containing or serum-free media and used for various clinical applications. The majority of cell-based therapeutics worldwide employ cells from single donors. Studies in the past have suggested inconsistencies in the therapeutic outcome of transplanting single donor-derived cells irrespective of the fact that they were haploidentical or HLA-mismatched^9^. Culture-expanded MSCs derived from different donors vary significantly in growth kinetics^10^, gene expression pattern^11,12^, anti-inflammatory, immunosuppressive activities^13–15^, and growth factor secretion profile^16^. These differences can be due to the sex, age, health status, and genetic makeup of the individuals. This heterogeneity poses a great challenge in determining the efficacy of MSCs in therapy and leads to mixed response in the outcome. Another major limitation in using single-donor MSCs is their limited ability to proliferate beyond a certain limit, precluding the production of the needed dose for therapeutic purpose. Further, it may be difficult to find MSCs from a second donor with identical therapeutic properties to the first donor from which the therapeutic products are developed. One possible way to overcome this hurdle is to pool the cells from different donors, possibly decreasing heterogeneity and also compensating for the deficits present in the individual donors. Pooled cell populations will have representation from different donors and have a cumulative advantage over individual donor MSCs. Further, pooling of cells from multiple donors increases the product size available for commercial use.

In this study, we analysed the effect of pooling MSCs from different donors on the growth kinetics, differentiation potential, phenotypic marker expression, colony forming unit-Fibroblast (CFU-F), senescence, in vitro tumorigenicity, secretome profile, and immunomodulatory potentials by comparing to the individual donor MSCs. For this purpose, we extracted MSCs from nine (9) different donors, culture expanded them, and characterised them for the abovementioned parameters. For the pooling study, the MSCs from three different donors were pooled at equal proportions at passage 1 (P1) and expanded till P5. We created three different pools, and the characterisation of the pooled cells were done at P3 and P5 and compared to the corresponding individuals. Our results suggests that pooling does not affect any of the basic qualities of MSCs and that the pooled cells exhibit broader, stable cytokine secretion and immunomodulatory profile in addition to reducing the heterogeneity of individual donor MSCs.

## Results

### Morphology and proliferation kinetics of individual and pooled BMMSCs

**Figure 1:**
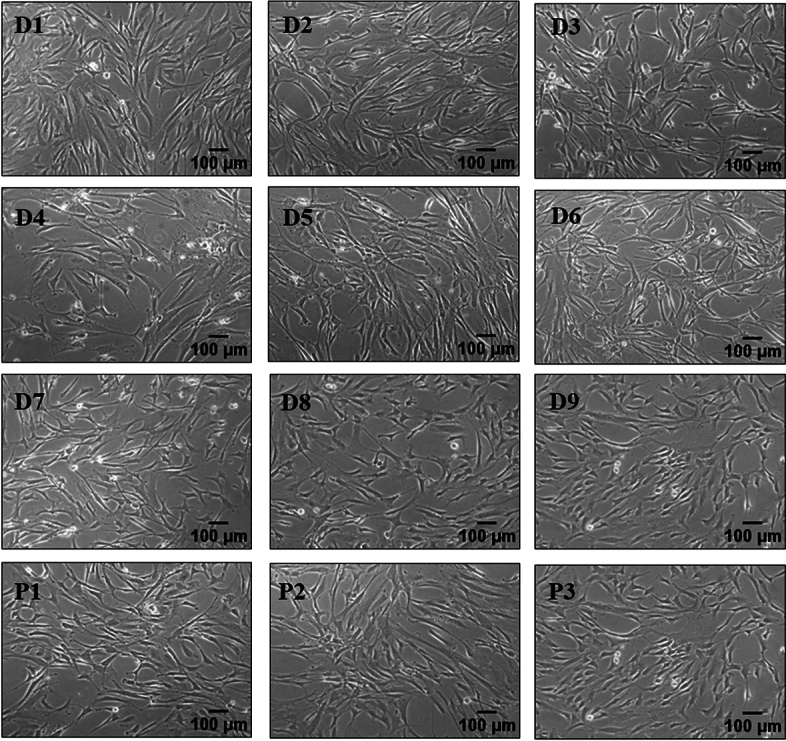
Morphological characteristics. Images of individual and pooled BMMSCs at passage 5 showing spindle-shaped morphology (magnification: 10x); D1–D9: Individual donors; P1–P3: Pooled cells (Scale bar: 100 µm).

**Figure 2:**
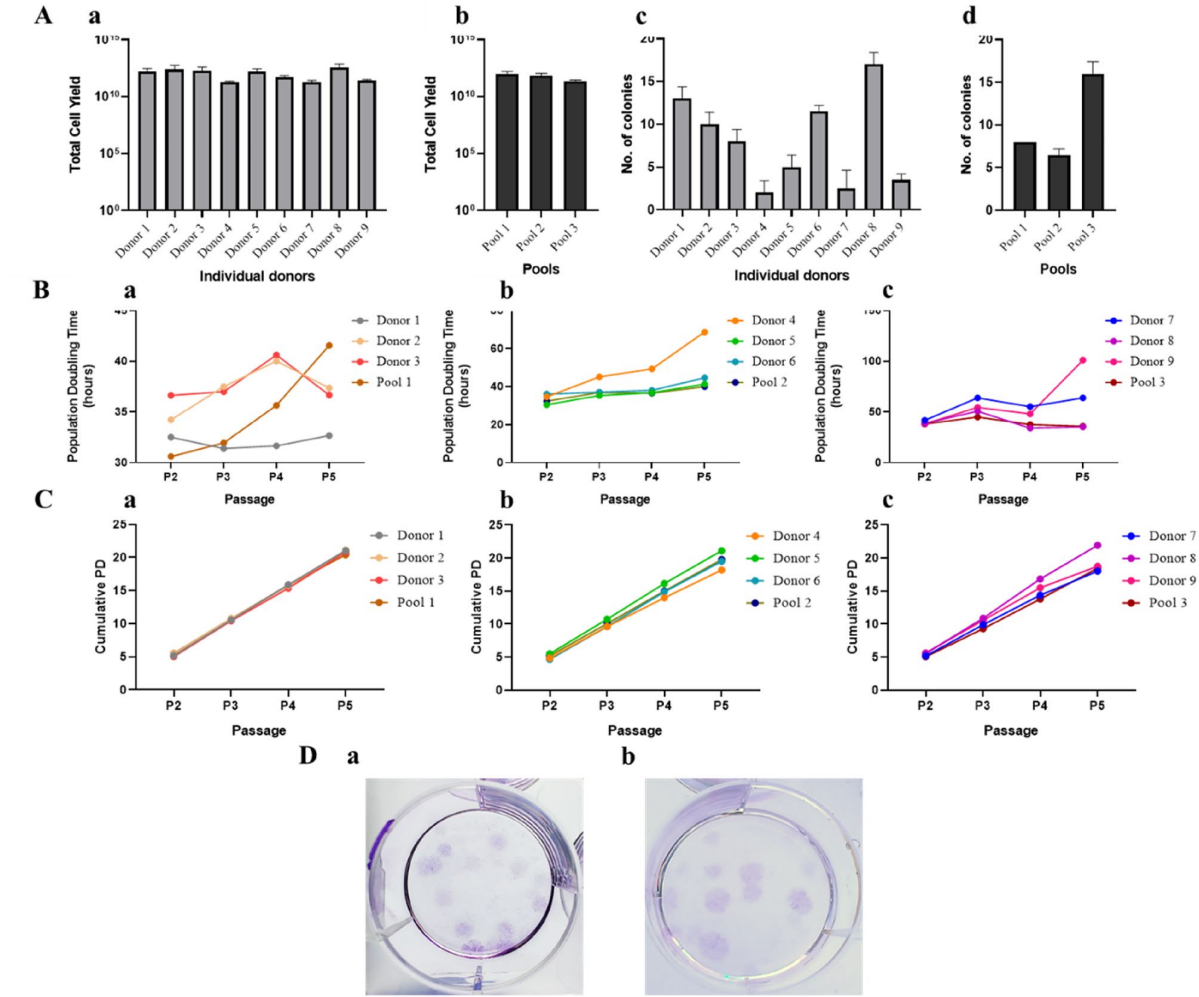
Proliferation kinetics. **A**(**a**, **b**)—Total cell yield of individuals and pools; **A**( **c**, **d**)—Colony forming unit-fibroblast (CFU-F) of individual and pooled samples; **B**(**a**, **b**, **c**)—Population doubling time of Pools 1, 2, and 3 compared to their individual donors; **C**(**a**, **b**, **c**)—Cumulative population doubling of pools 1, 2, and 3 compared to their individual donors. Marked heterogeneity observed in terms of cell yield, PDT, and CFU-F ability among the individual samples. The % CVs for pools are comparatively lower than that for the individuals for the proliferation kinetics; **D**(**a**, **b**)—Representative image of CFU-F colonies from 100 seeded cells of individual and pooled MSCs respectively.

We created a total of nine individual donor (Supplementary Table 1) BMMSCs and banked them as MCBs at P1. Three pools were created by mixing three randomly selected different individual donor MCBs in equal proportion. All the individual donor and pooled BMMSCs were expanded till P5 and cryopreserved at every passage. All the individual donor and pooled MSCs were spindle shaped at all the passages (Fig. 1). The proliferation kinetics in terms of total cell yield, population doubling time, and cumulative population doubling of these individual donor MSCs were measured from P2 to P5. The total cell yield of these individual MSCs varied from donor to donor and ranged from 1.9 × 10^11^ to 3.7 × 10^12^ cells (Fig. 2Aa). The pooled MSCs showed a comparatively homogenous cell yield profile (2.3 × 10^11^–9.9 × 10^11^) with a % CV of 60 than that of 90% in individuals (Fig. 2Ab, Supplementary Tables 2,3). The pooled MSCs had lower cell yield compared to the average of individual MSCs, but the difference was not significant. The population doubling time (PDT) at P5 was very heterogeneous among the individuals, varying from 33 to 101 h with an average time of 51 h (Fig. 2Ba–c, Supplementary Table 2. The pooled cells’ PDT was more aligned with each other, with an average time of 39 h (CV of 7% compared to 44% in individuals) (Supplementary Table 3). The cumulative population doubling (CPD) among the individual and pooled MSCs were similar, with an average of 20 till P5 (Fig. 2Ca–c ).

### Colony forming unit-Fibroblast (CFU-F) potential

CFU-F represents the colony forming abilities of the MSCs. All the individual MSCs formed colonies but with a heterogeneous CFU-F potential. The number of colonies varied from 2 to 17 (Fig. 2Ac,). The pooled MSCs also formed colonies but with the higher number of colonies compared to the individual MSCs (Fig. 2Ad). Though the average number of colonies formed by pooled MSCs was higher than that for the individual samples (10 vs. 8), the difference was not significant. The representative colony images are shown in Fig. 2Da,b

### Phenotypic marker expression

**Figure 3:**
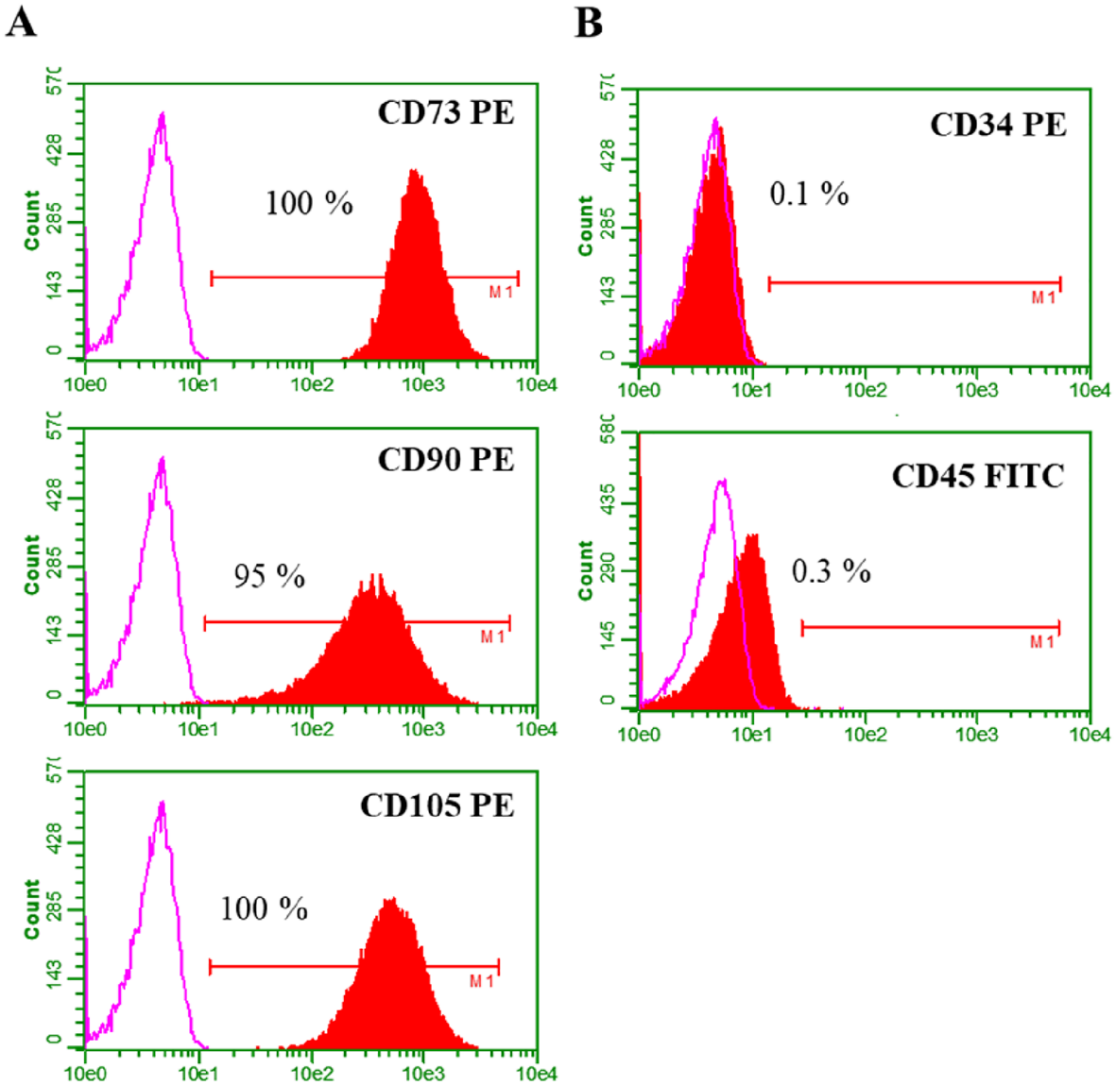
Phenotypic marker expression. Representative histograms of (**A**) positive marker and (**B**) negative marker expression by BM-MSCs. Both the individual and pooled MSCs showed expression of greater than 85% for positive markers and less than 1% for negative markers.

The expression of MSC-positive markers (CD73, CD90, CD105) and -negative markers (CD34, CD45) was analysed in both the individual and pooled MSCs. There was no significant difference in the expression of markers between the two populations. Both populations expressed the positive markers at a frequency of over 85% and negative markers at less than 1% (Fig. 3, Supplementary Table 4).

### Trilineage differentiation

**Figure 4:**
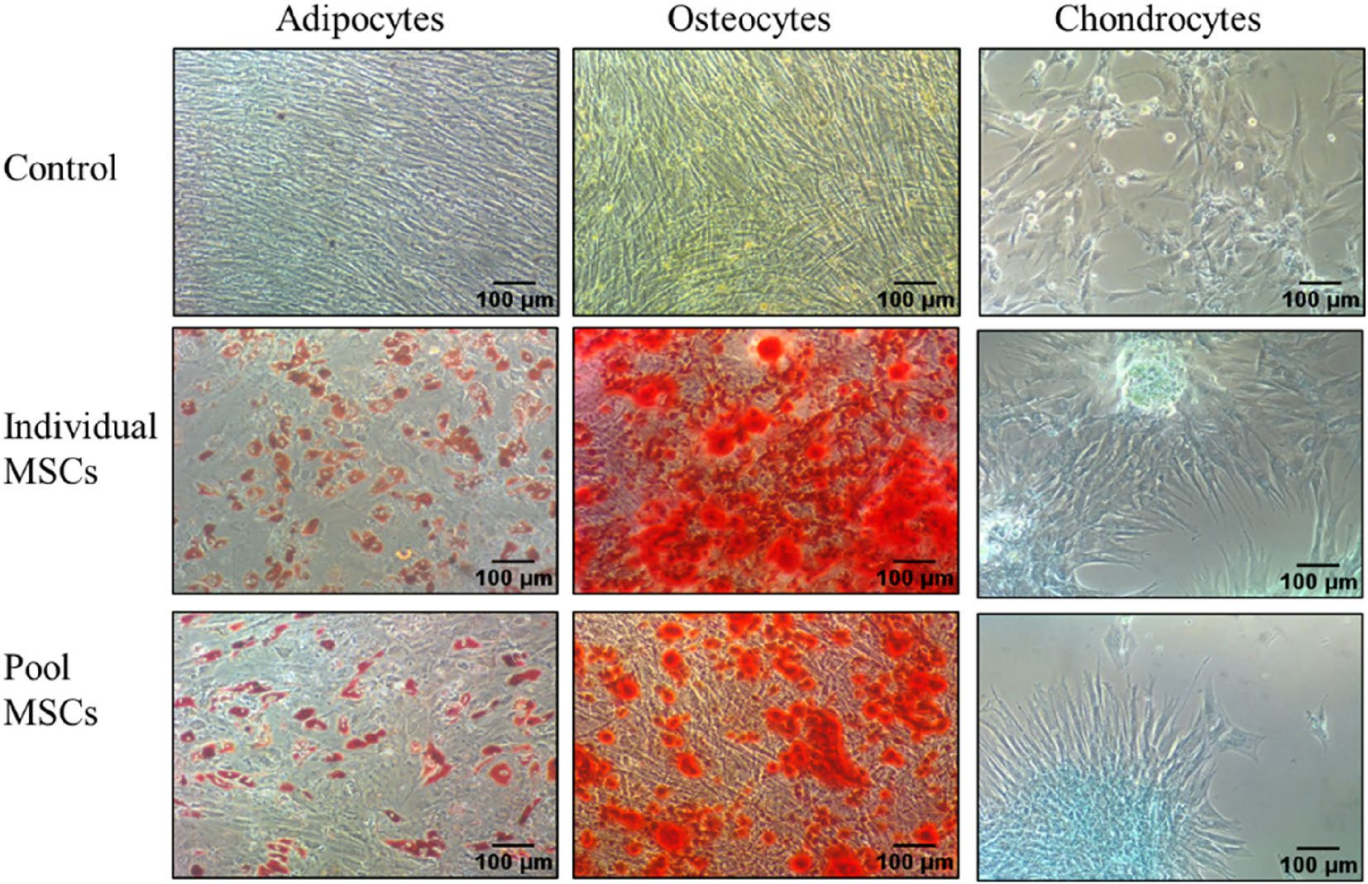
Trilineage differentiation. BMMSC differentiation into adipocytes, osteocytes, and chondrocytes as visualized by Oil Red O, Alizarin red, and Alcian blue stains, respectively, by representative individual donor and the pooled MSCs against undifferentiated controls (Scale bar: 100 µm).

The ability of the MSCs to differentiate into adipocytes, osteocytes, and chondrocytes was analysed at P5. When these cells were cultured in appropriate conditions, they differentiated into cells of all the three lineages, as confirmed by oil droplet staining with Oil Red O, calcium deposit with Alizarin Red, and chondrogenic mass with Alcian Blue (Fig. 4). The trilineage differentiation ability was preserved in both the individual and pooled MSCs and was not compromised by pooling. However, this was only qualitative data and was not used for comparison of individual and pooled cells.

### Immunosuppression

**Figure 5:**
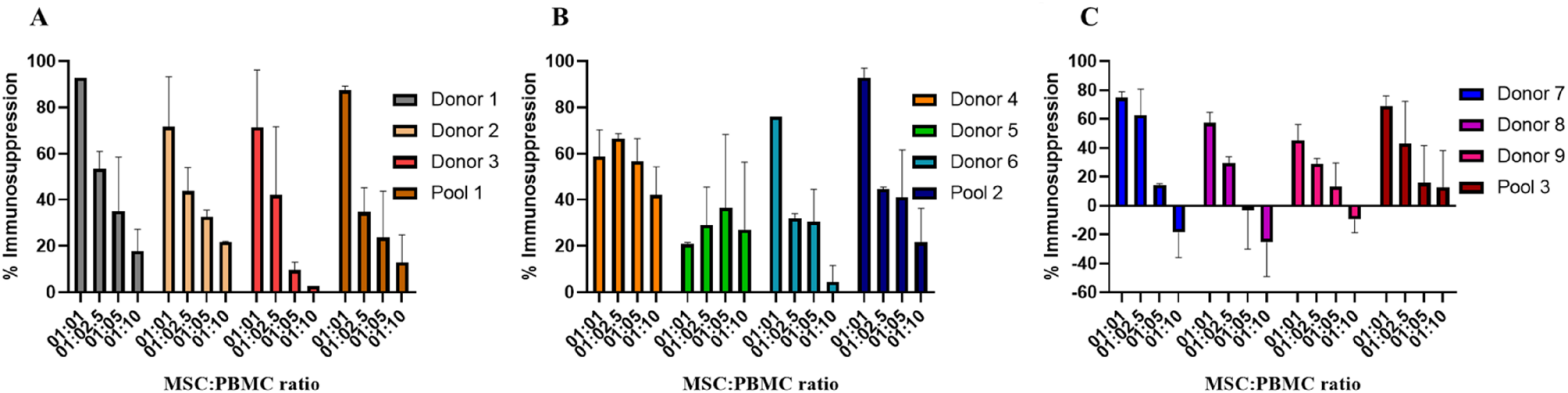
Immunosuppression. (**A**), (**B**), (**C**) Immunosuppressive capacity of Pools 1, 2, and 3, respectively, against their corresponding individual samples at different MSC:PBMC ratios. Pooled samples show consistent immunosuppression at all the ratios, while some individual MSCs failed to show immunosuppression at the 1:5 and 1:10 ratios.

We analysed the ability of MSCs to supress the proliferation of activated T cells in different MSC-to-PBMC ratios. The suppression percentages were compared between the individual donors to the corresponding pooled cell population. The individual and pooled MSCs showed immunosuppression at varying levels at the analysed ratios of 1:1, 1:2.5, 1:5 and 1:10. At the highest concentration of 1:1 ratio, the individual sample’s immunosuppression capabilities varied between 21 and 93% with % CV of 30 (Fig. 5A–C, Supplementary Table 5). At the lowest MSC concentration of 1:10, some of the individual samples failed to show immunosuppression. The heterogeneity was most pronounced at the ratio of 1:10, with a % CV of 300. On other hand, the pooled cells showed immunosuppression at all the ratios. At the highest ratio of 1:1, the percentage of suppression by pooled cells was greater than the average of corresponding three individual samples’ mean suppression; 88% versus 79% in pool 1, 93% versus 52% in pool 2, and 69% versus 59% in pool 3. Further, it is important to note that the pooled cells showed more homogenous profiles of immunosuppression with 15% CV at 1:1 and 32% CV at 1:10 ratios (Supplementary Table 6).

### Secretome of angiogenic factors

**Figure 6:**
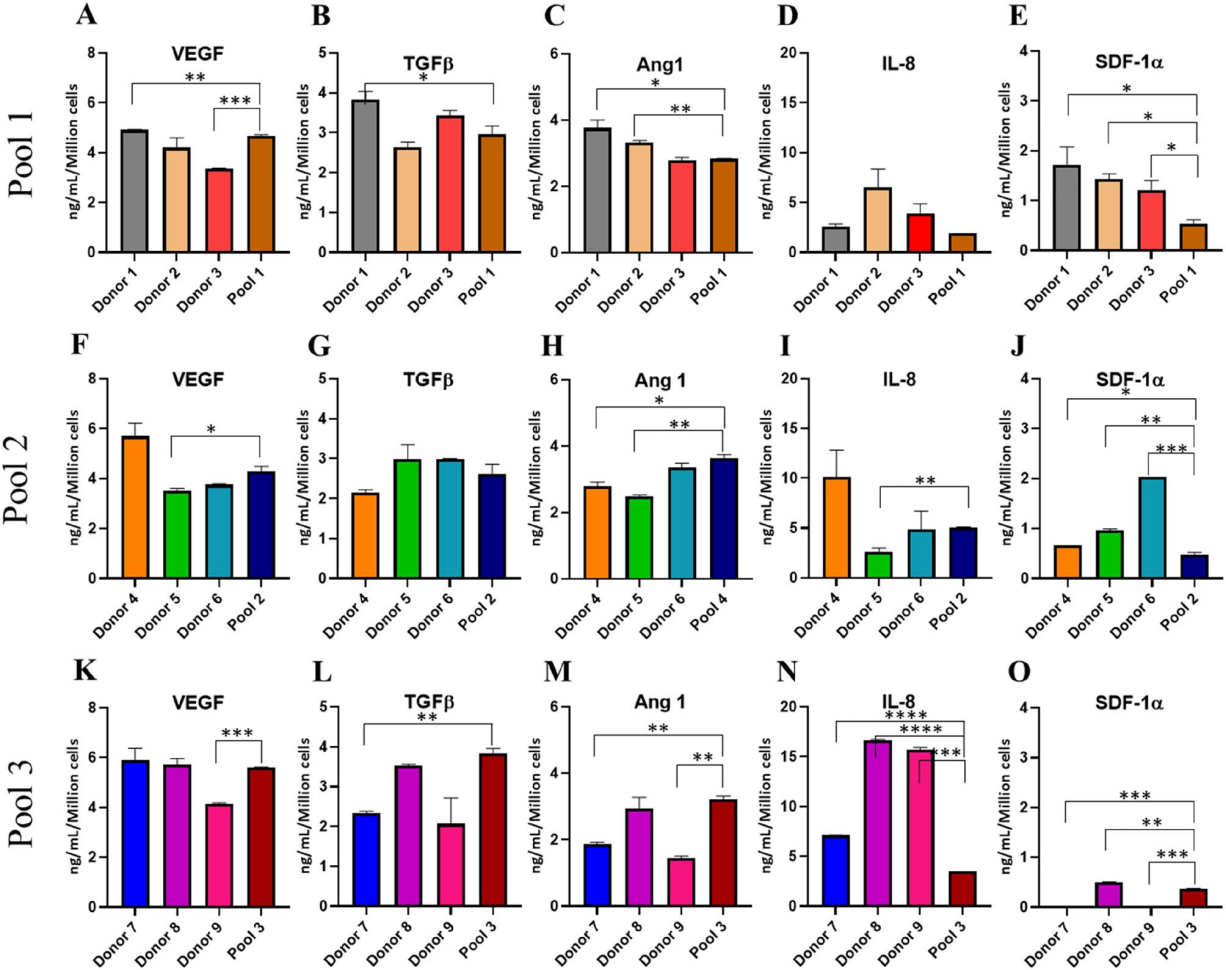
Secretome analysis. Bar diagram showing the expression levels of VEGF, TGFβ, Ang 1, IL-8, and SDF1α in individual samples and pooled MSC 1 (**A**–**E**), pooled MSC 2 (**F**–**J**), and pooled MSC 3 (**K**–**O**). The individual samples secreted more of IL-8 and less SDF-1. The pooled MSCs secreted higher amount of VEGF and lesser SDF-1. The heterogeneity in the secretion of these factors was seen more in individual samples compared to that in the pooled samples.

MSCs are known to secrete various angiogenic factors; thus, we analysed the amount of VEGF, TGFβ, ANG-1, IL-8, and SDF-1 in the conditioned media (Fig. 6A–O). The percentage coefficient of variation for these different cytokines varied from 20% for cytokines like VEGF to 71% for SDF-1 among the individual samples. On average, the individual MSCs secreted more IL-8 and less SDF-1, with some individual samples not secreting the latter at all. Noteworthy is that there was no single donor that secreted the highest amount of all these factors. Interestingly, the three pooled MSCs secreted all the analysed factors with % CV varied from 16 and 51% for VEGF and SDF-1 respectively. Further, the pooled samples secreted higher amount of VEGF, TGFβ, and ANG-1 and lesser IL-8 and SDF-1 compared to the average secreted by individual samples, though the values were statistically not significant.

### Senescence and in vitro tumorigenicity assay

**Figure 7:**
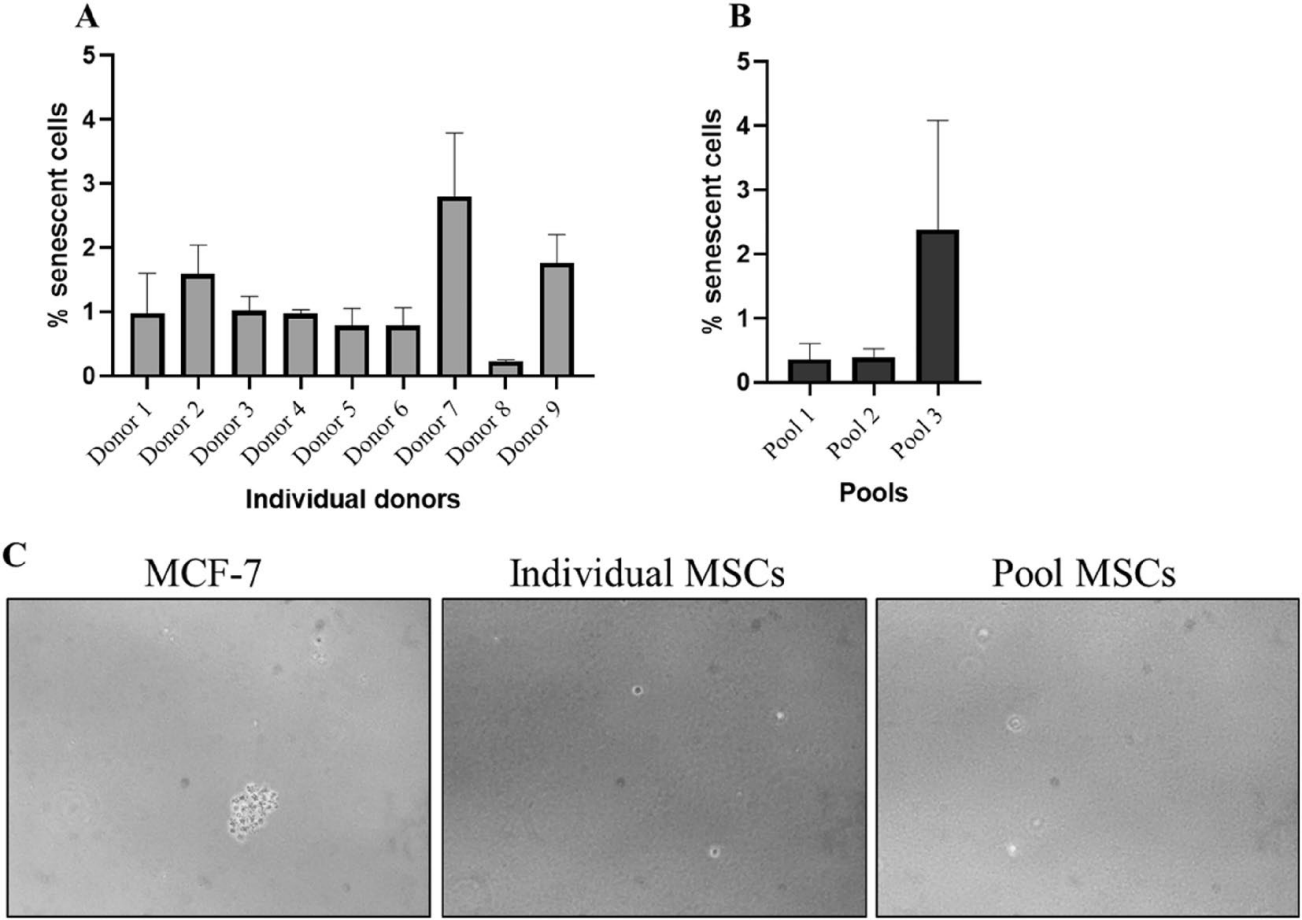
Senescence and in vitro tumorigenicity assay. (**A**, **B**)—Number of senescent cells in individuals and pools, respectively, at passage 5. **C**—Soft agar assay showing colonies in MCF-7-positive control and no colonies in either individual or pooled MSCs (Scale bar: 100 µm).

We analysed the percentage of MSCs that underwent replicative senescence at P5 by using senescence-associated β-galactosidase staining. We observed that there were very few senescent cells in the individual and pooled samples at P5 culture (Fig. 7A,B). The average proportion of senescent cells was less than 1.5% for both the individual- and pool-derived MSCs. The highest percentage was observed in donor 7 (2.8%) and in the corresponding pool 3 (2.4%). However, these values were negligible. We also checked for any transformation acquired during the cultures of these cells by culturing them in soft agar. Any transformed cells can grow in absence of solid matrix and will form colonies in soft agar. However, none of the individual or pooled MSCs formed any colonies compared to the MCF7 cancer cells that formed visible colonies (Fig. 7C).

### STR analysis

**Figure 8:**
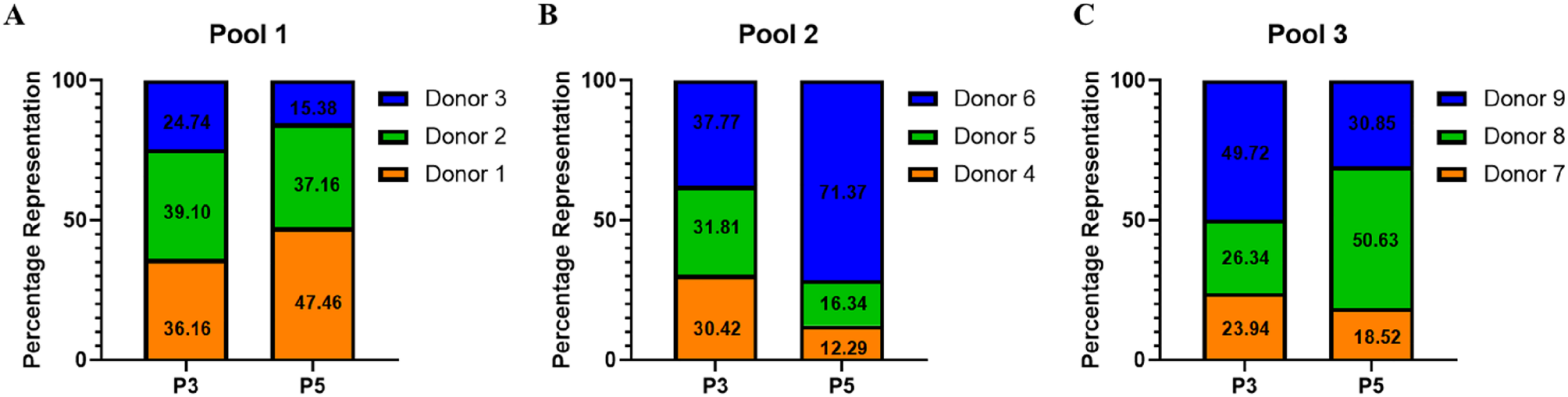
STR analysis. Percentage representation of different individuals in pools 1, 2, and 3 (**A**–**C**) at P3 and P5. As the passage progress, the dominance of a single donor becomes evident.

STR analysis done at the WCB stage revealed a more uniform representation of individual donors in pools 1 and 2, whereas in pool 3, one of the donors represented 50% of the pool while the other two donors had equal representations. At the P5 stage, we could still identify the presence of all the donors in the pools but with varying proportions. The least presentation of the individuals were found to be at 15%, 12%, and 18.5% in pools 1, 2, and 3, respectively (Fig. 8). The highest individual representations were at 47.5%, 71%, and 51% in pools 1, 2, and 3, respectively.

## Discussion

In clinical settings, MSCs are proven to be safe, but the efficiency outcome is mixed for different indications. As a result, there are very few MSC-based cell therapy products available commercially. One of the main reasons that contributes to this ambiguity is the heterogeneity of the MSCs, attributed to the innate biological variability of the donors^17–19^. Traditionally, MSCs are isolated from single donors, culture expanded, and cryopreserved, and after extensive characterization, the cells are used in clinical settings. There are several disadvantages of single donor-derived MSCs including the secretome profile not meeting the specifications, compromised immune regulatory properties, absence of differentiation potential to a particular lineage, batch-to-batch variation in specifications including potency assay, etc.^17,20–22^. Further, to obtain adequate cell therapy doses, single-donor MSCs either do not suffice in terms of volume or need to be expanded for a prolonged time in culture that contributes to poor proliferation potential and compromise the efficiency of the final product^23^.

One of the major hindering factors in cell therapy manufacturing is the heterogeneity of the product from different donors that leads to lack of reproducibility and unpredictable clinical outcome^19,24^. We developed a method for minimizing the heterogeneity and reproducibly producing an MSC product by pooling the MSCs from different donors in GMP conditions. Although previous reports mention pooling of different donors as a way to reduce heterogeneity^14,25^, there is no comprehensive study that has analysed different pools for characteristics like proliferation, differentiation, phenotypic marker expression, immunomodulatory potential, secretome, senenscence, and tumorigenicity of the pooled MSCs. Our study’s novelty relies on analyzing this entire range of parameters, including potency markers, between the individual and pooled MSCs. Another novelty of our study is the use of different pools comprising different biological replicates, leading to improved statistical strength of this study. Our previous reports also used this pooled MSC to prove their safety and efficiency in different preclinical and clinical settings^6,26–28^.

For this study, we isolated MSCs from nine different healthy, volunteer donors, expanded them, and banked them as MCBs at P1. For pooling, three different donor MCBs were mixed in equal proportion, expanded till P3, and banked at WCBs. The three pools created were cultured further till P5, and all further analyses were done on these cells.

The individual donor-derived MSCs and the pooled MSCs showed typical spindle-shaped morphology. There was no distinction in the morphology as both the individual and pooled cells looked similar. Further, pooling did not compromise the MSC characteristic phenotypic marker expression. Both the individual and pooled MSCs had positive marker expressions > 90%, while < 1% expression was noted for negative markers except for that in one single donor MSC that expressed CD90 at 85%. However, there was a reduced expression in the positive marker as per ISCT criteria^2^ as the expression should be ≥ 95%, but this minimum criteria is heavily debated, with amendments suggested by many groups^29^. Nevertheless, as the other ISCT criteria of plastic adherence and trilineage potential were met, of all our individual and pooled cells were indeed MSCs.

For chracterisation of proliferation, we analysed the total cell yield, PDT, and CPD of individual and pooled MSCs. Although unintentional, the donors in our study were all males between the ages of 22 and 29 years. It is worth noting that even with this small difference in the age among the donors, there was a large difference in the cell yield (CV = 90%). Similar differences on proliferation kinetics have been reported by others and was attributed to the age and sex of the donors^10,30–32^. Interestingly, the three pooled MSCs showed comparatively uniform cell yield, rather than the individual MSCs. Regarding the PDT, we observed a marked heterogeneity at P5 among the individual samples, with some individual donor PDT being double the average. Earlier studies also reported increasing doubling time in AD-MSCs with the passage number^33^. On comparison of average doubling time of individuals to their corresponding pools, the pooled MSCs had lesser doubling time, but the results were not statistically significant at all the passages. The pooling has shown a definite advantage in that even the pool (Pool 1) containing the slowest growing individual had a PDT similar to that of the other two pools. The CPD shows a more uniform trend among the individuals and pools, with the average CPD of individuals matching those of their corresponding pools. As CPD is one of the deciding factors of MSCs therapeutic potential, all our individual and pooled MSCs fell within the range of 20 CPDs^34^ and these cells were grown to the maximum of five passages as the efficiency was questionable beyond this^35^. For all these parameters (cell yield, PDT, and CPD), the CV for technical replicates was less than 10%, and we also observed that among these parameters the CPD showed the least intra-assay variability.

One of the other hallmarks of MSCs is their ability to form colonies. This was analysed using the CFU-F assay. Although all individual donor MSCs showed CFU-F capability, we found a large heterogeneity in the number of colonies formed by the individuals depending on their proliferation potential. Siegel et al.^30^ reported a similar finding in the variation in CFU-F potential among MSCs from different donors. Therefore, the pooling did not compromise the CFU-F ability; moreover, the pooled cells showed higher proliferation potential and number of colonies than the individual ones, though the results were not statistically significant.

Next, we evaluated the immunosuppressive capabilities of individual and pooled MSCs as this property plays a vital role in determining the efficacy of MSCs in treatments related to inflammatory and immune diseases. In the in vitro settings, immunosuppression was measured by the ability of the MSCs to suppress the proliferation of mitogen- or antigen-stimulated T lymphocytes or NK cells or B cells. In our study, we analysed the potential of individual and pooled MSCs to regulate the proliferation of phytohemagglutinin-stimulated PBMCs at different ratios ranging from 1:1 to 1:10 of MSC-to-PBMCs. This ratios were well within the range as proposed by the ISCT working proposal^36^ and also included a larger concentration of MSCs to analyse the maximum effect. As expected, we found a direct correlation between MSC and PBMC concentrations, with the maximum suppression obtained at a 1:1 ratio. However, we observed a large variation in the immunosuppressive abilities among the individuals at the different ratios (Supplementary Table 5). The % CV varied between 30 and 300% among the individual samples, with the highest difference observed at the 1:10 ratio. The intra-assay variability was also the highest in the ratio of 1:10 and least in the highest ratio of 1:1. It is also interesting to note that, some of the individual samples failed to show immunosuppression at the 1:1 and 1:10 ratios. As per the ISCT working proposal recommendations^36^, these donors are not eligible for the treatment of immune-related disorders. Interestingly, pooling of MSCs from three donors showed higher and more consistent immunosuppression, with % CV of 13% at 1:2.5 and up to 50% at 1:5 ratios (Supplementary Table 6). Similar to that in individual samples, the intra-assay variability was more prominent in 1:1 and least in the 1:10 ratio. This consistency of immunosuppression occurs, even with low or week immunosuppressive individual donor MSC’s were pooled and cultured together. It is also noteworthy that at the highest ratio, the immunosuppression of the pools was above the average values of the corresponding individual samples, though the results were not significant. Thus, pooling eventually reduced the heterogeneity among the individuals, and, due to some synergy that occurred by mixing the individuals, the pools showed consistent and better immunosuppressive profiles and could reduce the product variability. A similar result on pooling that decreases the intra-individual variability was reported earlier^15^.

To further understand if pooling has any distinctive benefit over the secretion of different cytokines, we analysed the set of angiogenic factors in the secretome of pools and their corresponding individuals. It is well known that MSCs secrete various angiogenic factors like VEGF, TGFβ, ANG-1, HGF, SDF-1 etc., and as a complex they are involved in neoangiogenesis and tissue regeneration^37^. Based on our previous studies^38,39^, we selected five angiogenic factors, viz., VEGF, TGFβ, ANG-1, IL-8, and SDF-1, that play major roles in the outcome of angiogenic diseases. The individual and the pooled MSCs secreted these five factors at varying concentrations in the culture. There was a large variation in the amount of these cytokines secreted by the individual donor MSCs. In the individual donor MSCs, on average, the factor IL-8 was secreted in higher concentrations followed by VEGF, with the least being SDF-1, and some donors not secreting SDF-1 at all. However, in case of pools, a more homogenous secretion pattern was observed compared to that in the individuals. All these five factors were secreted by the pooled samples, with VEGF being predominantly secreted and SDF-1 the least. This result is of importance when using the MSCs for treating vascular disorders since in our earlier studies we proved VEGF as a single important factor that determine the angiogenic potential of BMMSCs^38,39^. Another advantage of pooling is that the pools have consistent secretion of growth factors as in case of pool 3, which contains two out of three donors that do not secrete SDF-1 but still show a secretion level similar to that of the other pools. For all these factors analyzed, the inter-assay variability was less than 10% CV for both the individuals and the pools. It is also of note that among all these assays, secretome analysis showed the least batch to batch variability.

One of the main hurdles in cell therapy is that the cells reach replicative senescence in culture. As the senescent MSCs have decreased differentiation and immunomodulatory potentials that severely hinder the clinical outcomes of using MSCs^40^, it is important to analyse the percentage of senescent cells in the final cell therapy product. Since our product was the P5 MSCs, we examined the cultures for the presence of any senescent cells by using β-galactosidase staining. The percentage of senescent cells was less than 3% at P5, and even till P7 it was not more than 10% (data not shown) in both individual and pooled MSCs. It was also reported that MSCs in culture start to increase in size starting from P5^41^, but in our study we never noticed any size enlargement at P5. We confirmed that pooling does not have any impact on the morphology of the MSCs by inducing senescence at higher passages.

To understand if the observed effect of pooling was due to the presence of all the three individual donors, we performed STR analysis at the P3 and P5 stages. To our knowledge there are no previous studies that confirm the presence of individual donors in the pooled population. We found that all the three donors were present in the final pooled product but with different proportions. At P3, the representation of the individuals were uniform, but this ratio skews with some individual donor MSCs dominating in the pool at P5. We did not find any correlation between the dominant donors and the growth kinetics, i.e., it is not the highest yielder or the fast proliferator that is represented in higher percentage or vice versa. But interestingly there was a correlation between the individual donor CFU-F capacities and its final presence in the pooled population. In each of the pools, the dominant donor is the one with highest CFU-F. To have a better understanding, further studies can be planned by choosing individual donors with the same CFU-F and pooling them.

The major risk of using this as a therapy is the safety of stem cells with respect to their tumorigenic potential or their ability to form tumors after injection. The in vitro colony formation assay or soft agar assay is most commonly employed method to assess the tumorigenic potential of cell therapy products^42^. This method relies on the ability of transformed cells to grow in an anchorage-independent manner and form colonies in the soft agar. We noticed that pooling did not cause any transformation in the MSCs as observed by the absence of colonies in both the individuals and pooled MSCs. A previous report from our lab also confirmed this finding^32^.

Despite being a comprehensive study, there are still questions providing scope for future studies. All our analyses are conducted at P5, raising questions about whether the properties of the pooled cells change after this passage as the dominance of single donor become more and when other donors get eliminated from the pool. Also, for our pooling experiment we used only 3 donors as the tracking would be comparatively easier. We also did not explain the effect of pooling if more than 3 donors form a pool. Further, all the donors in our study were males, and the future studies must be done using all female donors and mixed donors to check the potential effects of sex on pooling. The current study can be r strengthened by analysing the global gene expression pattern of the individuals and the corresponding pools.

## Conclusion

From this study, we propose a solution to the issue the current cell therapy field is facing. The outcome of cell therapy is often mixed and difficult to predict, and it is contributed to the heterogeneity of the cellular medicinal products. To make a consistent MSC-based cell therapy product, we pooled MSCs from three different donors at the P1 stage and banked them at P3 as working cell banks. These WCBs were the starting materials to have a reliable and reproducible final pooled product at P5. Our pooled cells showed all the basic characteristics of typical MSCs and expressed superior immunosuppression characteristics along with stable secretion of angiogenic factors. They also did not exhibit any senescence in culture and were non-tumorigenic. These pooled cells were also proven to be safe and effective and could increase the number of product sizes for different preclinical and clinical settings.

## Methods

### BMMSC isolation and culture conditions

The bone marrow donation was undertaken from voluntary donors of age between 18 and 40 years after obtaining their informed consent at Kasturba Hospital, Manipal, Karnataka, India. The study protocol has been approved by Manipal Academy of Higher Education (MAHE) Ethics Committee, Manipal, Karnataka, India (Reg No.: ECR/191/Inst/KL/2013/RR-19). The confidentiality of the donor had been maintained. The data has been reported accurately and have no conflict of interest. All methods were performed in accordance with the relevant guidelines and regulations. The bone marrow samples were filtered through 100-µm cell strainer to remove any bone spicules and mixed in equal proportion with medium containing Knockout Dulbecco’s modified Eagle’s medium (KO-DMEM) and 10% fetal bovine serum (FBS). The samples were centrifuged at 300 g for 10 min at room temperature to remove any anticoagulants. The pellet was resuspended in medium containing KO-DMEM with 10% FBS and overlaid above the lymphoprep solution (Alere Technologies). The buffy coat was separated out by differential centrifugation and washed once with above said media. The extracted cells were expanded in complete media containing 88.5% KO-DMEM (Gibco-Invitrogen), 10% FBS (Hyclone), 1 × GlutaMAX I (100x- Gibco), and 0.5% penstrep (Gibco) in a 37 °C incubator at 5% CO_2._ The cells were passaged when they reached 80–90% confluency and cultured thereafter in complete media with 2 ng/mL basic fibroblast growth factor (bFGF) (Gibco).

### Banking and pooling strategy

The BMMSCs were banked in two tier system, with passage 1 (P1) cells of individual donors named the Master Cell Bank (MCB) and passage 3 (P3), the Working Cell Bank (WCB). For creation of pools, three individual donor MCBs at P1 were mixed in equal proportions and culture expanded till P3 and stored as WCBs. For the manufacturing of the final product, the pooled WCBs were further expanded for two more passages till P5 and cryopreserved. The three different pools along with their corresponding individual donors were analysed for various characteristic in triplicate and compared.

### Morphology and proliferation analysis

The morphological features of the cultured BMMSCs were analysed using 10X magnification in a bright field-phase contrast microscope (Nikon Eclipse TE2000-S), and the representative images were captured at every passage. The total number of cells harvested was counted using an automated cell counter, and the population doubling time (PDT) was calculated using the formula^43^:

$${\text{PDT }} = \, \left( {{\text{Log N}}_{{\text{t}}} {-}{\text{LogN}}_{0} } \right)/{\text{Log2}}$$

where N_t_ = Final cell yield during passaging. N_0_ = Initial no. of cells seeded.

### Colony forming unit-fibroblast (CFU-F) assay

The BMMSCs ability to grow by forming colonies was analysed using CFU-F assay. Cells were seeded at a density of 100 cells per well in 6 multi-well tissue culture treated plates in duplicates. The culture plates were maintained for 11 days in an incubator at 37 °C, 5% CO_2_ with complete media change every 3 days. Fixation was done with 1% paraformaldehyde (prepared in 1 × Phosphate-buffered saline (PBS) with pH 7.2–7.4) for 30 min at room temperature. Subsequently, it was washed with 1 × PBS and then stained for 1 h using 1% (w/v) crystal violet (HiMedia Laboratories). The excess stain was washed with distilled water, and the number of stained colonies were counted manually.

### Surface marker analysis

MSC-specific CD marker analysis was carried out for positive (CD73, CD90, CD105) and negative markers (CD34, CD45) using flow cytometry. The antibodies were either from BD Pharmingen or R&D systems. The antibodies CD 73, CD 105, CD 45 were used as neat concentration and CD90 at 1:10 and CD 34 as 1:50 dilution. Briefly, 1 × 10^6^ cells were incubated with the respective fluorescently labelled antibodies for 30 min at room temperature in 96-well micro-test plates (U bottom non-coated) for analysis. Samples were run in a Guava Easycyte Plus System flow cytometer and analysed using the Cytosoft 5.2 software.

### Trilineage differentiation

For analysing the potential of MSCs to differentiate into adipocytes, osteocytes, and chondrocytes, the BMMSCs were seeded at a density of 1000 cells/cm^2^ in 6 multi-well tissue culture dishes in complete media. At 60–70% confluency, differentiation was induced by adding the respective stem pro differentiation media (Invitrogen-Gibco). Cells were observed, and media was replenished every 3 days. At 21 days post-induction, differentiated cells were fixed with 4% paraformaldehyde (PFA) for 30 min and stained with the following solutions at room temperature. The staining of cells was performed with 0.5% Oil Red O stain prepared in 100% Isopropyl alcohol (IPA) for adipogenic differentiation, 1% Alizarin Red S prepared in distilled H_2_O for osteogenic differentiation, and 1% Alcian Blue prepared in 0.1N HCL for chondrogenic differentiation (All the stains from HiMedia Laboratories). Representative images were captured at 10 × magnification using a phase contrast microscope (Nikon Eclipse TE2000-S) with undifferentiated MSCs serving as a negative control.

### Immunosuppression assay

To understand the BMMSC’s ability to suppress the immune reaction, MSCs were co-cultured with activated peripheral blood mononuclear cells (PBMCs), and their proliferation was measured. The PBMC were isolated by lymphoprep density gradient centrifugation. Briefly, the peripheral blood obtained from healthy volunteers was diluted in plain RPMI medium and overlaid in lymphoprep at a 2:1 ratio. The mixture was centrifuged at 600 g for 15 min and the PBMCs in the buffy coat were carefully removed and washed once with the media. After isolation, a part of PBMC’s was activated with 10 µg/ml of Phytohaemagglutinin (PHA)^44^. The activated and naïve PBMCs were plated in 96 well plates, and above which Mitomycin C treated BMMSCs were plated at different densities to yield MSC:PBMCs ratios of 1:1, 1:2.5, 1:5, and 1:10. The cells were left undisturbed for 3 days after which 5-bromo-2-deoxyuridine (BrdU) was added and incubated for a further 24 h. The proliferation of PBMCs was measured following the BrdU proliferation kit (Roche) manufacturer’s protocol. Briefly, after 24 h, the BrdU was removed and fixative was added for 30 min. The anti-BrdU-POD was added after removal of fixative and incubated for 1 h. The wells were washed with washing solution, and the substrate was added for 30 min. Then the plates were read on an ELISA reader at 450 nm (reference wavelength: 690 nm) after adding the stop solution.

### Secretome analysis

To analyse the levels of specific cytokines or chemokines or growth factors in the conditioned medium secreted by the cells, ELISA kits (R&D Systems) were used according to the manufacturer’s protocols. Briefly, MSCs were seeded at a density of 1 × 10^6^ cells per T-75 flask in duplicates and grown in complete medium (with bFGF) for 72 h. The conditioned medium (CM) were collected from the flasks, centrifuged at 500 g for 5 min (to remove cell debris), filtered using a 0.22-μm syringe filter (Merck-Millipore), and analysed as per kit instructions.

### Senescence assay

The replicative senescence in MSCs was analysed using a senescence-associated β-galactosidase staining kit (Cell Biolabs). Briefly, cells were cultured in 6 multi-well plates till they reached confluency of around 60–70% and were then fixed/stained according to the manufacturer’s protocol. After overnight incubation, the cell-staining working solution was removed and washed twice with 1X PBS. Representative images were taken at 10 × magnification using a phase contrast microscope, and senescent cells were counted manually in each field. The percentage of senescent cells was derived by dividing the total number of senescent cells (stained) by the total number of cells (unstained).

### Soft agar assay

The in vitro tumorigenicity property of the MSC’s was studied using the soft agar assay method^45^. In 6 multi-well plates, 0.6% agarose (low EEO-Himedia) was added and allowed to solidify for 1 h inside a laminar hood. Above this layer, BMMSCs (8000 cells) mixed in 0.3% agarose were overlaid, and after solidification, 1 mL of complete media was added and maintained in a CO_2_ incubator at 37 °C. Media change was carried out every 3 days, and the cells were observed under a phase contrast microscope for the formation of any colonies. As a positive control, the cancer cell line MCF-7 was cultured simultaneously. After 28 days in culture, plates were stained with 0.1% crystal violet solution and images were captured under the microscope.

### Short tandem repeat (STR) analysis

The presence of individual donor MSCs in the final pooled population were analysed by detecting the DNA short tandem repeat (STR) regions in the individuals and comparing that to the corresponding pools. The DNA was extracted using a DNA extraction kit (Qiagen) from the individual and pooled MSCs at P3 and P5 stages. The DNA samples were subjected to multiplex STR Typing by AmpFlSTR^®^ Identifiler^®^, in which different alleles present in 16 STR loci (D8S1179, D21S11, D7S820, CSF1PO, D3S1358, TH01, D13S317, D16S539, D2S1338, D19S433, vWA, TPOX, D18S51, AMEL, D5S818, and FGA) were amplified in a single reaction, followed by capillary electrophoresis on a SeqStudio Genetic Analyzer. The most informative STRs were used to differentiate and quantify the individual donor presence in the pooled sample.

### Statistical analysis

All experiments were done in triplicate. GraphPad Prism software was used for analyses. Quantitative data are reported as mean ± standard deviation (SD). A *p* value of less than 0.05 was considered statistically significant.

### Supplementary Information

Supplementary Tables.

## Data Availability

All data generated is provided within the manuscript or supplementary information files.

## Abbreviations

ANG-1: Angiopoietin 1
AT: Adipose tissue
bFGF: Basic fibroblast growth factor
BM: Bone marrow
BrdU: 5-Bromo-2-deoxyuridine
CD: Cluster of differentiation
CFU-F: Colony forming unit fibroblast
CO_2_: Carbon dioxide
CM: Conditioned medium
CPD: Cumulative population doubling
CV: Coefficient of variation
EEO: Electroendosmosis
ELISA: Enzyme-linked immunosorbent assay
FBS: Fetal bovine serum
hBMMSCs: Human bone marrow mesenchymal stromal cells
GTG banding: Giemsa banding
GvHD: Graft versus host disease
HCL: Hydrochloric acid
HLA: *Human leukocyte antigen*
IL-8: Interleukin-8
IMP: Investigational medicinal product
IPA: Isopropyl alcohol
ISCT: International society for cellular therapy
KO-DMEM: Knockout Dulbecco’s modified Eagle’s medium
MCB: Master cells bank
MCF-7: Michigan cancer foundation-7
MSC: Mesenchymal stromal cells
MNCs: Mononuclear cells
NK: Natural killer
PBS: Phosphate-buffered saline
PBMC: Peripheral blood mononuclear cells
PDT: Population doubling time
PFA: Paraformaldehyde
PHA: Phytohaemagglutinin
SD: Standard deviation
SDF-1: Stromal cell-derived factor 1
STR: Short tandem repeat
TGF-β: Transforming growth factor β
VEGF: Vascular endothelial growth factor
WCB: Working cell bank
