# Supplementary material for: Advantages of pooling of human bone marrow-derived mesenchymal stromal cells from different donors versus single-donor MSCs
Source: Sci Rep. 2024 Jun 2;14:12654. doi: 10.1038/s41598-024-62544-8 (PMC11144708; doi:10.1038/s41598-024-62544-8)
Supplement: Supplementary file 1 — Supplementary Tables. [file 41598_2024_62544_MOESM1_ESM.docx]

Supplementary Table 1- Age and sex of individual donors

| Donor Id | Age | Sex |
| --- | --- | --- |
| Donor 1 | 28 | Male |
| Donor 2 | 23 | Male |
| Donor 3 | 26 | Male |
| Donor 4 | 26 | Male |
| Donor 5 | 28 | Male |
| Donor 6 | 22 | Male |
| Donor 7 | 29 | Male |
| Donor 8 | 26 | Male |
| Donor 9 | 29 | Male |

Supplementary Table 2- Growth kinetics of individual donor-derived BMMSCs

Figure 1. Morphological characteristics

| Parameters | Donor 1 | Donor 2 | Donor 3 | Donor 4 | Donor 5 | Donor 6 | Donor 7 | Donor 8 | Donor 9 | Mean | SD | % CV |
| --- | --- | --- | --- | --- | --- | --- | --- | --- | --- | --- | --- | --- |
| Total cell yield (in millions) | 1.68E+12 | 2.43E+12 | 1.91E+12 | 1.93E+11 | 1.67E+12 | 5.00E+11 | 1.80E+11 | 3.68E+12 | 2.77E+11 | 1.39E+12 | 1.2E+12 | 90 |
| CPD in days | 21.04 | 21.11 | 20.83 | 18.19 | 21.11 | 19.50 | 18.04 | 21.95 | 18.76 | 20.06 | 1.5E+00 | 7 |
| Doubling time in hours at P5 | 32.67 | 37.37 | 36.67 | 68.72 | 41.29 | 44.62 | 63.76 | 35.24 | 101.01 | 51.26 | 2.3E+01 | 44 |

Supplementary Table 3- Growth kinetics of Pooled BMMSCs

| Parameters | Pool 1 | Pool 2 | Pool 3 | Mean | SD | % CV |
| --- | --- | --- | --- | --- | --- | --- |
| Total cell yield (in millions) | 9.88E+11 | 6.61E+11 | 2.34E+11 | 6E+11 | 4E+11 | 60 |
| CPD in days | 20.40 | 19.79 | 18.47 | 2E+01 | 1E+00 | 5 |
| Doubling time in hours at P5 | 41.56 | 39.95 | 35.96 | 4E+01 | 3E+00 | 7 |

Supplementary Table 4- Percentage of phenotypic marker expression

| CD markers | Donor 1 | Donor 2 | Donor 3 | Donor 4 | Donor 5 | Donor 6 | Donor 7 | Donor 8 | Donor 9 | Pool 1 | Pool 2 | Pool 3 |
| --- | --- | --- | --- | --- | --- | --- | --- | --- | --- | --- | --- | --- |
| CD73 | 100 | 100 | 99.9 | 100 | 99.7 | 99.7 | 99.9 | 100 | 100 | 100 | 100 | 100 |
| CD90 | 95.4 | 99.5 | 97.1 | 96.2 | 98.5 | 98.7 | 85.9 | 99.5 | 96.3 | 93.7 | 94.7 | 94.8 |
| CD105 | 100 | 100 | 100 | 99.6 | 99.9 | 99.7 | 99.9 | 100 | 99.6 | 99.9 | 99.9 | 100 |
| CD34 | 0.1 | 0.2 | 0.2 | 0.3 | 0.2 | 0.2 | 0.2 | 0.2 | 0.2 | 0.2 | 0.4 | 0.3 |
| CD45 | 0.3 | 0.3 | 0.2 | 0.3 | 0.9 | 0.9 | 0.3 | 0.2 | 0.4 | 0.3 | 0.8 | 0.4 |

Supplementary Table 5- Immunosuppression profiles of individual donor-derived BMMSCs

| MSC: PBMC Ratio | Donor 1 | Donor 2 | Donor 3 | Donor 4 | Donor 5 | Donor 6 | Donor 7 | Donor 8 | Donor 9 | Mean | SD | % CV |
| --- | --- | --- | --- | --- | --- | --- | --- | --- | --- | --- | --- | --- |
| 1:1 | 92.86 | 71.64 | 71.33 | 58.76 | 20.89 | 76.10 | 74.90 | 57.37 | 45.18 | 63.23 | 20.88 | 30 |
| 1:2.5 | 53.36 | 43.75 | 42.07 | 66.55 | 29.16 | 32.02 | 63.05 | 29.67 | 28.89 | 43.17 | 14.81 | 30 |
| 1:5 | 35.20 | 32.66 | 9.60 | 56.74 | 36.45 | 30.64 | 14.18 | -3.28 | 13.16 | 25.04 | 18.12 | 70 |
| 1:10 | 17.90 | 21.78 | 2.75 | 42.04 | 27.20 | 4.54 | -18.61 | -25.37 | -9.72 | 6.95 | 22.31 | 300 |

The values in the table are the percentage of immunosuppression at different MSC: PBMC ratios and the negative value indicates absence of immunosuppression

Supplementary Table 6- Immunosuppression profiles of pooled BMMSCs

| MSC: PBMC Ratio | Pool 1 | Pool 2 | Pool 3 | Mean | SD | % CV |
| --- | --- | --- | --- | --- | --- | --- |
| 1:1 | 87.47 | 92.91 | 69.01 | 83.13 | 12.53 | 15 |
| 1:2.5 | 34.61 | 44.60 | 42.77 | 40.66 | 5.32 | 13 |
| 1:5 | 23.71 | 41.20 | 15.67 | 26.86 | 13.05 | 50 |
| 1:10 | 12.99 | 21.63 | 12.71 | 15.78 | 5.07 | 32 |
